# A qualitative and quantitative assessment of cardiac cine Phase contrast MRI: comparison of image quality between 2D and 3D acquisition

**DOI:** 10.1186/1532-429X-17-S1-P54

**Published:** 2015-02-03

**Authors:** Munemura Suzuki, Takahiko Nakazono, Ken Yamaguchi, Kiyoshi Dogomori, Yoshiaki Komori, Norihiko Kotooka, Koichi Node, Hiroyuki Irie

**Affiliations:** 1 Radiology, Saga University Hospital, Saga, Japan; 2 Suzuki Medicalimaging Labo, Kagoshima, Japan; 3 Cardiology, Saga Universuty Hospital, Saga, Japan; 4 Research & Collaboration Department, Siemens Japan K.K., Tokyo, Japan

## Background

3D and gapless multi-slice 2D cine Phase contrast (PC) MRI enables the visualization and analysis of 4D (3D + time) intracardiac flow. However, the image quality which affects the accuracy of segmentation of left ventricle (LV) has not been discussed. In this study, we compared the magnitude image and the source image of PC MR angiography (MagSUM) derived from both 2D and 3D PC MRI. For the qualitative analysis, we assessed the image quality for the delineation of endocardial border. And we measured LV volume and compared with standard cine MRI for the quantitative analysis.

## Methods

Ten healthy volunteer (all male, median age: 24 years) underwent 2D and 3D prospectively ECG-gated PC MRI with respiratory naviation on 3T MRI (Trio a Tim, Siemens AG Healthcare Sector, Erlangen, Germany).The short axial plane was employed to cover left ventricle. The common parameters for PC MRI were: VENC = 150 cm/sec, segmentation factor = 2 to 3, reconstruction phase = 12 to 14 according to R-R interval. The acquisition voxel of 2D and 3D PC MRI were reconstructed into 2.1x2.1x5 mm and 2.1x2.1x2.1 mm, respectively. Standard 2D retrospectively ECG-gated cine MRI was reconstructed to 2.1x1.8x8 mm. Two readers discussed and determined the image quality for one short axial image (mid-ventricle level), and two reconstructed images (vertical and horizontal long axial image) of end-systolic, early- and mid-diastolic phase using 4 point scale (1 = poor, 2 = fair, 3 = good, 4 = excellent). One blinded reader manually measured the area of LV chamber and multiplied by the slice thickness and added together to obtain the end-systolic and mid-diastolic LV volume using PC MRI. Another blinded reader also measured cine MRI as reference standard. P<0.05 was regarded as significant.

## Results

**Figure 1:**
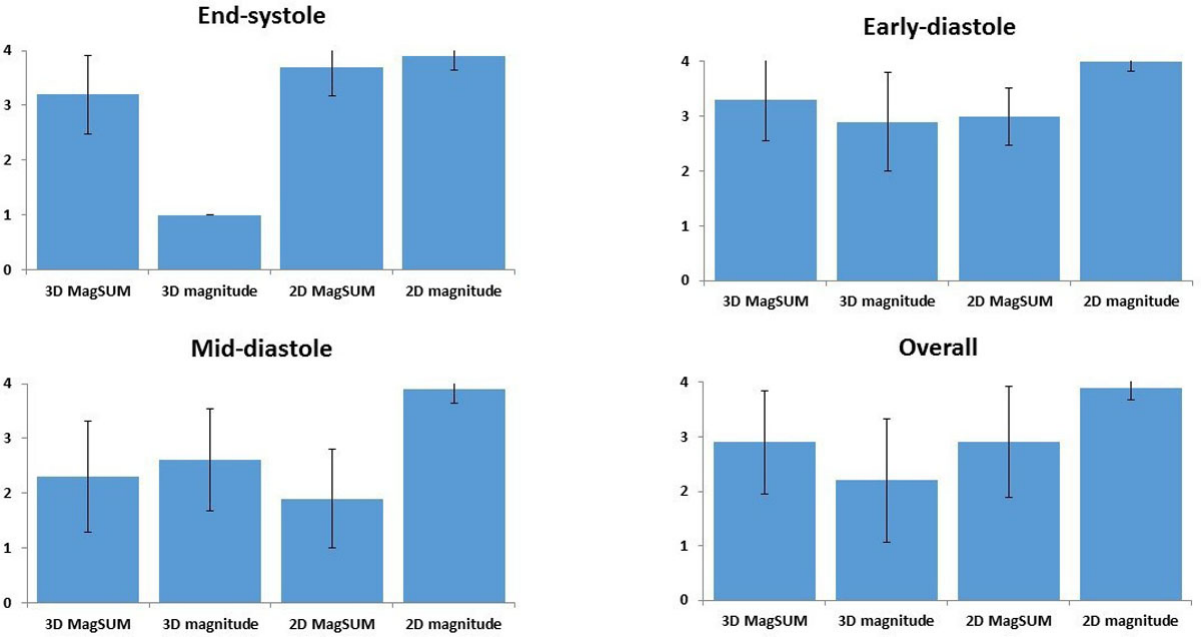
Quantitative analysis of image quality (4-point scale) for delineation of endocardial border (MagSUM: the source image of phase contrast MRI).

**Figure 2:**
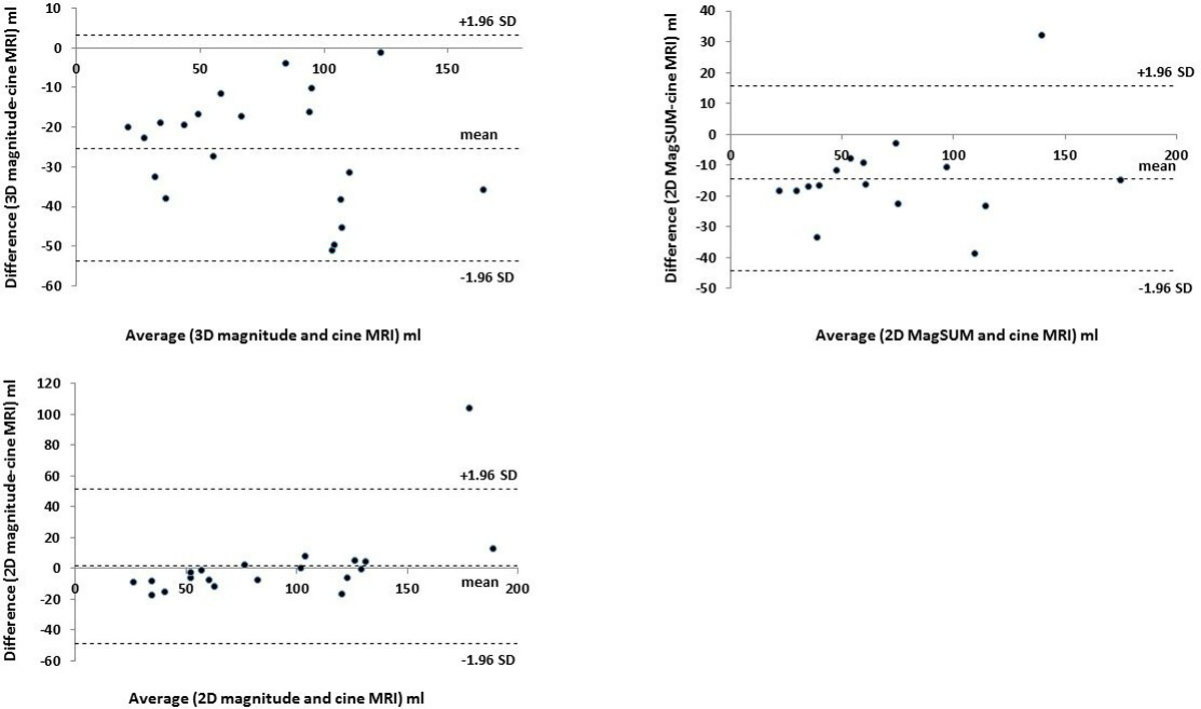
Bland-Altman Plot for left ventricular volume measured by phase contrast MRI and standard cine MRI.

The magnitude image of 2D PC MRI provided the best image quality. We could not recognize the endocardial border in 3D magnitude image at end-systolic phase in all case and excluded from quantitative analysis (Figure 1). Four 2D MagSUM images were also excluded because of significant artifacts. Strong correlation of LV volume were seen between PC MRI and cine MRI (3D MagSUM, 2D MagSUM: r = 0.94 and 2D magnitude: r = 0.91) although the 2D and 3D MagSUM images systemically underestimated (Figure 2).

## Conclusions

The magnitude image of 2D PC MRI seems to provide accurate segmentation of LV. We have to pay attention when using MagSUM image of 2D and 3D PC MRI for LV segmentation.

## Funding

N/A.

